# Bat4RCT: A suite of benchmark data and baseline methods for text classification of randomized controlled trials

**DOI:** 10.1371/journal.pone.0283342

**Published:** 2023-03-24

**Authors:** Jenna Kim, Jinmo Kim, Aejin Lee, Jinseok Kim

**Affiliations:** 1 School of Information Sciences, University of Illinois Urbana-Champaign, Champaign, Illinois, United States of America; 2 Department of Statistics, University of Illinois Urbana-Champaign, Champaign, Illinois, United States of America; 3 Institute for Social Research, University of Michigan, Ann Arbor, Michigan, United States of America; 4 School of Information, University of Michigan, Ann Arbor, Michigan, United States of America; University of Southern California, UNITED STATES

## Abstract

Randomized controlled trials (RCTs) play a major role in aiding biomedical research and practices. To inform this research, the demand for highly accurate retrieval of scientific articles on RCT research has grown in recent decades. However, correctly identifying all published RCTs in a given domain is a non-trivial task, which has motivated computer scientists to develop methods for identifying papers involving RCTs. Although existing studies have provided invaluable insights into how RCT tags can be predicted for biomedicine research articles, they used datasets from different sources in varying sizes and timeframes and their models and findings cannot be compared across studies. In addition, as datasets and code are rarely shared, researchers who conduct RCT classification have to write code from scratch, reinventing the wheel. In this paper, we present Bat4RCT, a suite of data and an integrated method to serve as a strong baseline for RCT classification, which includes the use of BERT-based models in comparison with conventional machine learning techniques. To validate our approach, all models are applied on 500,000 paper records in MEDLINE. The BERT-based models showed consistently higher recall scores than conventional machine learning and CNN models while producing slightly better or similar precision scores. The best performance was achieved by the BioBERT model when trained on both title and abstract texts, with the F1 score of 90.85%. This infrastructure of dataset and code will provide a competitive baseline for the evaluation and comparison of new methods and the convenience of future benchmarking. To our best knowledge, our study is the first work to apply BERT-based language modeling techniques to RCT classification tasks and to share dataset and code in order to promote reproducibility and improvement in text classification in biomedicine research.

## 1. Introduction

As the number of biomedical research articles rapidly increases, extracting relevant information from the massive scientific literature has become a non-trivial task for researchers who want to mine biomedical texts for learning and discovering knowledge in medicine, pharmaceuticals, and biology. In particular, randomized controlled trials (RCTs) are being conducted, making existing information retrieval methods challenged in automatically distinguishing whether a research article is related to RCT or not. The RCT is a study in which people are randomly allocated to receive one of clinical interventions such as treatment [1]. RCTs are used to examine the effect of interventions on specific outcomes such as death or the recurrence of disease and play a major role in guiding clinical research as well as real-world practices. As a result, the demand for highly accurate retrieval of RCT information in scientific articles has grown. However, identifying all published RCTs in a given domain is not simple. Research has shown that only approximately 85% of RCT-related papers in MEDLINE, a bibliographic database including peer-reviewed journal papers in biomedicine (https://www.nlm.nih.gov/medline/medline_overview.html), are annotated with the publication type ‘Randomized Controlled Trial’ [2], suggesting that many RCT papers are not annotated and thus excluded from literature review and analyses by scholars.

Manually assigning a RCT tag to a research paper is not sustainable considering the increasing pace of research papers in biomedicine. As a result, researchers have attempted to automate RCT tagging using machine learning approaches. As linear‐kernel support vector machines (SVM) gained popularity in text classification [3], for example, several studies have used SVM models to predict whether a research article is an RCT study or not. In Cohen et al. [2], SVM was trained on a large subset of MEDLINE and achieved the area under a receiver operating characteristic (ROC) curve of 0.973 and mean squared error of 0.01. Thomas et al. also used a SVM classifier trained and tested on the Cochrane crowd dataset and produced a very high recall of 0.99 but a very low precision of 0.08 [4]. Wallace et al. tried to make the process of RCT classification more efficient using a hybrid approach of crowdsourcing and machine learning [5]. They trained a SVM classifier on the EMBASE dataset to filter research articles that are deemed unlikely to be RCTs and defer uncertain cases to crowd-sourced workers for manual tagging. The results of their experiment showed 95%–99% recall with less effort than relying on manual screening alone. Lanera et al. tried to train a SVM classifier on a PubMed (https://pubmed.ncbi.nlm.nih.gov/) dataset that can identify RCT-related research articles with a comparable word characterization in the ClinicalTrials.gov clinical trial repository [6]. Table 1 provides an overview of the prior studies that identify RCTs with their main models and datasets. It also compares the characteristics of our dataset to existing ones. It is worth noting that despite the diversity of data sources, sizes, text types, and reported accuracies, the prior research heavily relies on using SVM and CNN models for RCT classification.

**Table 1:** Overview of prior work to identify RCTs in the biomedical literature and comparison of our dataset with existing ones. Despite the diversity of data sources, sizes, text types, and reported accuracies, the prior research heavily relies on SVM and CNN models.

Citation (Year)	Corpus	Data Type	Data Size (Training/Test)	Data Ratio (RCT/Non-RCT)	Model	Performance
Cohen et al. (2015) [2]	PUBMED	Abstract	5,483K/834K	1:21.7	SVM	F1 0.824 Precision 0.873 ROC 0.973
Wallace et al. (2017) [5]	EMBASE COCHRANE	Abstract	111K/47K	1:23.2	SVM	Recall 0.71 precision 0.99
Marshall et al. (2017) [8]	COCHRANE CLINICAL HEDGES	Abstract & Title	280K/49K	1:6.0	CNN	Recall 0.957 precision 0.559 ROC 0.984
Lanera et al. (2018) [6]	PUBMED	Abstract & Title	1.7K/234K	1:37.2	SVM	Sensitivity 0.999 Specificity 0.972–0.999 ROC 0.934–0.999
Fiol et al. (2018) [9]	PUBMED CLINICAL HEDGES	Abstract & Title	363K/31K	1:1.7	CNN	Recall 0.969 Precision 0.346
Thomas et al. (2020) [4]	EMBASE CLINICAHEDGES COCHRANE	Abstract & Title	281K/44K	1:11.8	SVM	Recall 0.99 Precision 0.08
Chen et al. (2021) [10]	ORAL SCIENCE (Chinese)	Abstract & Title	10K/2K	1:1.6	CNN	ROC 0.99
**Our dataset**	PUBMED	Abstract & Title	490K/50K	1:4	LR, SVM, GB, CNN, BERT	Accuracy 0.964 Recall 0.902 Precision 0.922 F1 0.909

Other researchers have used deep learning to assign RCT tags to biomedical research articles as neural network models have been reported to outperform conventional machine learning models in classification tasks [7]. For example, Marshall and colleagues trained and optimized SVM and Convolutional Neural Networks (CNN) models on the titles and abstracts of the Cochrane Crowd RCT set [8]. Their CNN model achieved recall of 95.7 with precision of 55.9, demonstrating that deep learning can produce better performance in RCT classification than widely used machine learning models such as SVM. Fiol’s team trained a CNN classifier using 403,000 PubMed citations with title and abstract, and compared their model’s prediction results with search filters, such as McMaster’s textword search [9]. Their deep learning model obtained similar recall (96.9% vs 97.1%) and higher precision (34.6% vs 22.4%) than the search filter. In [10], a CNN model was trained on 12,166 Chinese papers published in the area of ‘Oral Science’ and produced a promising performance with the area under a ROC curve of higher than 0.99.

In this study, we investigate how pre-trained language models can be adopted for RCT classification tasks. Our study is motivated by two considerations. First, deep learning approaches, especially pre-trained language models, have demonstrated outstanding performance in many natural language processing (NLP) tasks. In the general domain, it was shown that unsupervised pre-training of language models on a large corpus, followed by fine-tuning of the models for a particular task, can improve the performance of many NLP algorithms, including text classification [11,12]. Although a few studies showed that early neural networks models such as CNN can outperform conventional machine learning models in RCT classification [8–10], recently developed contextual language models, BERT (Bidirectional Encoder Representations from Transformers) [13] or its variants, have never been applied to RCT classification tasks. In this work, we present Bat4RCT (Baseline Tool for RCT classification), an integrated method to serve as a strong baseline for RCT paper classification and a dataset to be used for implementation. We design a comprehensive framework that includes the application of BERT-based language modeling techniques in addition to conventional machine learning approaches on biomedical text data for predicting RCTs.

Second, although existing studies have provided invaluable insights into how RCT tags can be predicted for biomedicine research articles, they have datasets from different sources in varying sizes and timeframes (years). As a result, even if many of them used the same classifiers (SVM and CNN), their models and findings cannot be compared across studies. In addition, other models than SVM and CNN have rarely been compared with the performance of selected models. To address these issues, our approach explores a variety of BERT-based models such as BERT, BioBERT (BERT for biomedical text mining), and SciBERT (BERT for scientific text). We successfully trained and validated these models on a large-scale subset of research articles randomly selected from the entire MEDLINE database. For comparison, we also implemented three conventional machine learning models (Logistic Regression (LR) [14], SVM, and Gradient Boosting (GB) [15]), one neural networks model (CNN [16]), and one simple heuristic (keyword match) to gauge performance gains or losses of BERT-based models and integrated those models into our proposed system of data and code, Bat4RCT.

Our main contributions are as follows:

- We propose a strong baseline method, which is a part of Bat4RCT, to evaluate how well a research article which is related to RCT can be predicted using the state-of-the-art pre-trained language models in comparison with conventional machine learning techniques.
- We evaluate the baseline method in Bat4RCT on a large-scale dataset containing records spanning 10 years and release the dataset to encourage further research in the field of RCT classification.
- We summarize the characteristics of datasets and models used for prior work to identity RCT papers in the biomedical literature and compare them with our study.
- We open-source the code and dataset for future evaluation and comparison of new methods and benchmarking (https://github.com/jennak22/Bat4RCT).

To the best of our knowledge, this study is the first to employ BERT-based language modeling techniques to RCT classification tasks and to share the dataset and code in order to promote improvement in text classification in biomedicine research.

The organization of the paper is as follows. Section 2 describes the data collection and presents descriptions of the models and techniques used for RCT classification in this work. It also provides details about our experimental approach. In Section 3, we analyze the obtained results. Section 4 discusses the results. Finally, in Section 5, we conclude and discuss avenues for future work.

## 2. Materials and methods

### 2.1 Dataset

Data were collected by querying the MEDLINE database, which is one of the U.S. National Library of Medicine (NLM) literature archives that curate metadata of more than 33 million research articles in biomedicine. A public release (November 2021 version) of the entire MEDLINE baseline was downloaded from the NLM’s repository and parsed into files in SQL format for information retrieval. Next, any research article with ‘Randomized Controlled Trial’ as ‘publication type’ was identified and its unique document identifier (PMID) was recorded. Among research articles published over 2011–2020, 100,000 PMIDs were randomly selected for RCT articles and 400,000 PMIDs for non-RCT articles. Any PMIDs that include null values for either title or abstract texts were excluded from the selection. In our collection, a small number of PMIDs appeared multiple times with updated information. Only the most recent ones among duplicates were selected for retrieval. Lastly, title and abstracts were extracted for the selected PMIDs. Since this study is a binary classification task (RCT vs. non-RCT), each PMID instance is assigned a class label of either 1 (RCT) or 0 (non-RCT) based on its publication type information. The final dataset contains a total of 499,963 instances with five attributes (PMID, pubtype (class label), year, title, and abstract text), which is then randomly split into the subsets of training, validation, and testing using a fixed random seed, which contain 80%, 10%, and 10% of the data, respectively.

### 2.2 Experiments

This study aims to compare the performance of a variety of RCT-text classification methods that serve as a competitive baseline approach for RCT classification research. For this purpose, three conventional classifiers (LR, SVM, and GB) and a deep learning method (CNN) were chosen as they represent high performing methods in many classification tasks. As a BERT-based approach is introduced as a strong contender in this research, three BERT-based models were employed: BERT, BioBERT, and SciBERT. We also added a simple heuristic approach to compare the performance of the algorithmic methods mentioned above (see Section 2.2.4 Heuristic approach). Each model was trained on the training set with three types of texts: title, abstract, and combined title and abstract. The validation set was used for CNN and BERT-based models to identify the best performing approaches. The selected best BERT models were tested using the test set and their performance was compared to those of a heuristic method, three conventional classifiers, and the CNN model.

#### 2.2.1 BERT-based models

BERT is a contextual word representation model learned from large-scale language model pre-training of a bidirectional transformer [13]. Developed by Google, BERT is based on the transformer architecture trained on data from Wikipedia and the Book Corpus containing more than 10,000 books of different genres. Following pre-training on general natural language corpora, BERT can readily be fine-tuned for many NLP tasks such as named entity recognition and Question & Answering by using small datasets that show high performance. Variants of BERT have been adapted to specific domains by pre-training on specialized corpora such as biomedical literature. For this study, we used an English-language BERT-base cased model which contains 12 transformer blocks, 768 hidden layers, 12 self-attention blocks, and 110 million parameters in total. BioBERT is a domain-specific BERT based model trained on large-scale biomedical data from PubMed and PubMed Central (https://www.ncbi.nlm.nih.gov/pmc/) in addition to the corpora of the original BERT [17]. BioBERT has boosted the enhanced performance on many biomedical NLP tasks such as named entity recognition, biomedical relation extraction, question answering, etc. BioBERT-Base v1.1 was used for our study. SciBERT follows the same architecture as BERT but is pre-trained on scientific text instead [18]. It is trained on 1.14M papers extracted from the Semantic Scholar (https://www.ncbi.nlm.nih.gov/pmc/) [19] that cover biomedical and computer science domains. SciBERT was used for evaluation on different tasks such as named entity recognition, sentence classification, and dependency parsing with datasets from a variety of scientific domains, showing meaningful improvements over BERT on several of these tasks [18,20]. The version of the model used in this study is scibert-scivocab-cased.

#### 2.2.2 Pre-processing

We ran different BERT models to compare their results against traditional machine learning models. In NLP machine learning tasks, it is necessary to pre-process unwanted texts for feature extraction and selection. However, pre-trained language models such as BERT and its variations do not require such pre-processing tasks because they use those sequences and positions of words to understand the intent of user inputs. Thus, the following steps were implemented only for applying classical machine learning approaches. Our data went through four steps to normalize texts and remove elements that may affect classification negatively: (1) converting all letters into lowercase; (2) tokenizing sentences using the NLTK toolkit [21]; (3) removing punctuation and stop words to exclude words that do not have important meanings; and (4) stemming words using the Porter Stemmer in the NLTK to reduce the dimensionality of vocabulary. Once the data were cleaned, TF-IDF was used as a feature selection strategy that converted all string tokens into numerical values to be passed through classical machine learning algorithms.

For using the BERT-based models, we avoided any preprocessing steps other than tokenization. BERT uses a word-piece tokenizer that splits words in pieces where one word can be broken into multiple tokens [13]. As BERT variants are trained with texts that have been prepared differently, the choice of tokenizer is tied to the choice of BERT model. To process our input data, tokenizers for the three BERT-based models were loaded using the Huggingface transformers library [22]. Each input sentence was processed by the BERT specific tokenizer and then truncated to 512 tokens if it was from an abstract or combined abstract and title because BERT-based models can only process sentences of length with 512 tokens in maximum. For processing title texts, the maximum length was limited to 150 tokens. This limit was determined based on the distribution of entire tokens to handle the longest sentence.

#### 2.2.3 Implementation settings

We ran all our experiments on AWS (Amazon Web Services) SageMaker, a cloud machine learning platform that allows researchers to build and train models. We implemented conventional classification algorithms on the ‘ml.r5.2xlarge‘ instance type of SageMaker notebook using Scikit-learn (version 0.24.1), a Python machine learning library [23]. The parameters used for training all the conventional models were set to default. For instance, the LR model was fit with L2 regularization and a maximum threshold of 100 iterations for optimization. The GB classifier was trained using 100 estimators and a learning rate of 0.1.

For the CNN model, Keras (version 2.4.3) (https://keras.io/) and Tensorflow (version 2.3.4) (https://www.tensorflow.org/) were used for training and testing. Keras is a Python-based deep learning API that runs on the TensorFlow machine learning platform developed by Google [24]. A ‘ml.g4dn.2xlarge’ notebook instance type enabling NVIDIA Tesla T4 was used. GPU utilization reached the maximum usage of 14.7GB of memory for each training loop. For creating the CNN architecture, we followed the approach described in Fiol et al [9]. The first layer applied word embeddings built from the training data to capture semantic similarity. The tensors formed from this step were passed through two convolutional layers with kernel size of two and three, respectively. Each convolutional layer contains 512 filters and ReLU as an activation function. The output was passed through a max pooling procedure after a convolutional layer was applied. The resulting max-pooled values were then concatenated into a single layer. A dropout regularization of 0.5 was added to prevent overfitting. Results were passed to the fully connected final layer that uses Sigmoid to assign a probability of class belonging to either 1 or 0. To align with the environment for running BERT-based models, we used a batch size of 16 and ran the training loop for 4 epochs. Also, ADAM [25] and binary cross entropy were chosen for an optimizer and loss function, respectively. The maximum length of input for the embedding layer was set to 150 for title texts and 512 for both abstract texts and combined abstract and title texts.

We fine-tuned three BERT-based models with our training data using the Huggingface transformers library (version 4.15.0) (https://github.com/huggingface/pytorch-transformers) and PyTorch (version 1.7.1) (https://pytorch.org/) framework. The Huggingface transformers library provides a well-furnished API to leverage pre-trained deep learning models aimed at various NLP tasks. PyTorch is an optimized tensor library developed by Facebook’s AI research lab and is one of the widely used open-source deep learning frameworks. A ‘ml.p3.2xlarge’ instance type enabling NVIDIA Tesla V100-SXM2-16GB GPU was used for each SageMaker session for training and testing. The maximum usage of 15.7GB GPU of memory was reached for each training loop. We used a learning rate to 2e-5 with a batch size of 16 and ran the training loop for 4 epochs, following the recommendations from the original BERT paper [13]. The maximum token length was set to 150 for title texts and 512 for abstract and combined title and abstract texts based on the distribution of words in the dataset. Optimization was done using ADAM, and binary cross entropy was chosen as the loss function. The model was checked against a validation set after every optimizing step.

#### 2.2.4 Heuristic approach

We used a simple keyword matching technique as a heuristic classifier for comparing with the performance of algorithmic models. After removing punctuation marks, a list of defined keywords was matched on texts and each text was labeled as 1 (RCT) if any of terms in the list was detected within the text regardless of letter case, and otherwise as 0 (non-RCT). Only exact match was considered and any text with at least 1 keyword of the list detected was labeled as 1. Table 2 shows a list of keywords used for the heuristic approach.

**Table 2:** A list of keywords used for the heuristic classification method.

Keyword	’RCT’, ’RCTs’, ’randomized controlled trial’, ’randomized controlled trials’, ’randomised controlled trial’, ’randomised controlled trials’, ’randomized trial’, ’randomized trials’, ’randomised trial’, ’randomised trials’, ’randomized clinical trial’, ’randomized clinical trials’, ’randomised clinical trial’, ’randomised clinical trials’, ’randomized controlled’, ’randomised controlled’, ’radomized clinical’, ’randomised clinical’, ’randomized’, ’randomised’, ’clinical trial’, ’clinical trials’, ‘controlled trial’, ’controlled trials’

## 3. Results

In this section, we describe the evaluation results of our classification models for RCT prediction. We report the results in terms of four metrics: accuracy, precision, recall, and F1. Accuracy indicates the proportion of correctly predicted texts among all prediction results. Precision is the proportion of correctly predicted RCTs among those predicted by a model as RCTs. Recall refers to the proportion of correctly predicted RCTs among all the correct RCTs. F1 score is the harmonic mean of recall and precision.

Precision, recall, and F1 measure are defined as follows:

- TP (True Positive): number of instances classified as a class and actually belonging to that class
- FP (False Positive): number of instances classified as a class and actually not belonging to that class
- FN (False Negative): number of instances belonging to a class but classified as a different class

Precision = TP ÷ (TP + FP)

Recall = TP ÷ (TP + FN)

F1 = (2 × Recall × Precision) ÷ (Recall + Precision)

### 3.1 Overall performance

Table 3 summarizes the evaluation results of the heuristic model, three conventional machine learning models, CNN, and three BERT-based models in predicting the RCT instances on the test set (50,000 instances). We observed that conventional ML models, CNN, and BERT-based models outperformed the heuristic for all combination types of text (title, abstract, title + abstract) with respect to almost all evaluation metrics. One exception was that the heuristic model obtained the highest precision on ‘title only’ text data (95.02%), which was expected as the heuristic method was designed to classify a title instance as RCT if it contains any of target words in the list. However, the simple matching method missed many title instances without any of the target words in them, producing the lowest recall score of 42.02%. This also explains why the heuristic method performed worst on abstract and combined title and abstract test sets that contain many instances where any of the target words were not present.

**Table 3:** Summary of evaluation results of a heuristic model, three conventional machine learning models (LR, SVM, and GB), a neural networks model (CNN), and three BERT-based models (BERT, BioBERT, and SciBERT) for using (a) title, (b) abstract, and (c) combined title and abstract as input data. Bold indicates the best performance results among all models. LR: Logistic Regression, SVM: Support Vector Machines, GB: Gradient Boosting, CNN: Convolutional Neural Networks.

**Method**	**Model**	**Accuracy (%)**	**Precision (%)**	**Recall (%)**	**F1 (%)**
Heuristic	Heuristic	87.97	**95.02**	42.02	58.28
Conventional Machine Learning	LR	91.54	86.54	68.34	76.37
	SVM	91.80	87.24	69.13	77.13
	GB	89.60	90.94	53.33	67.23
Neural Networks	CNN	92.01	86.37	71.28	78.10
BERT-based	BERT	92.05	83.39	75.25	79.11
	BioBERT	**92.96**	84.68	**79.10**	**81.79**
	SciBERT	92.87	84.70	78.53	81.50
(a) Title texts					
**Method**	**Model**	**Accuracy (%)**	**Precision (%)**	**Recall (%)**	**F1 (%)**
Heuristic	Heuristic	90.66	82.92	67.11	74.18
Conventional Machine Learning	LR	94.77	90.83	82.12	86.25
	SVM	95.12	91.34	83.50	87.24
	GB	94.98	91.66	82.42	86.79
Neural Networks	CNN	95.40	90.27	86.31	88.25
BERT-based	BERT	96.02	91.90	87.82	89.81
	BioBERT	96.23	91.36	**89.62**	90.48
	SciBERT	**96.28**	**91.97**	89.20	**90.56**
(b) Abstract texts					
**Method**	**Model**	**Accuracy (%)**	**Precision (%)**	**Recall (%)**	**F1 (%)**
Heuristic	Heuristic	91.78	83.70	73.14	78.06
Conventional Machine Learning	LR	94.93	91.40	82.42	86.68
	SVM	95.33	91.93	84.04	87.81
	GB	95.27	**92.16**	83.43	87.58
Neural Networks	CNN	95.29	**92.16**	83.55	87.64
BERT-based	BERT	96.27	91.97	89.11	90.52
	BioBERT	**96.37**	91.52	**90.18**	**90.85**
	SciBERT	96.36	92.01	89.60	90.79
(c) Combined (title + abstract) texts					

Among conventional models, SVM outperformed LR and GB in terms of recall and F1, but their performance gaps were within around a 1%-point difference in most cases. In terms of precision, GB produced the highest scores across all three types of test sets. The CNN model showed similar or slightly better performance than the conventional models and worse than the BERT-based models in terms of accuracy, recall, and F1 when trained on all the combination types of text. For precision, it outperformed the BERT-based models when using title and combined title and abstract texts, but produced the worst results among all the models except the heuristic for abstract texts.

Compared to the conventional ML models, all three BERT-based models showed similar performance in precision but outperformed overall (F1) due to the substantial gains (5%~20% point improvements) in recall. BioBERT gave the best performance with accuracy of 96.37%, recall of 90.18%, and an F1 score of 90.85%, when both title and abstract texts were used for training and testing. While BioBERT trained on combined title and abstract texts produced the highest score in recall, it showed similar performance (89.62%) when only abstract texts were trained. Across all three types of texts, the worst performing BERT model showed better results than any of the conventional classifiers with the highest performance in terms of accuracy and F1. In addition, the BERT-based models produced optimal results with much higher recall scores than traditional ML models, with more RCT texts being correctly predicted among the entire test data.

For predicting non-RCTs (i.e., Class 0), the BERT-based models outperformed the other models in terms of accuracy, precision, and F1 when trained on all input text types. Table 4, which follows, reports the results. Among the BERT-based models, BioBERT showed the best performance when using title and combined title and abstract texts. For recall, the BERT-based models did not show much improvement compared to other models. The highest score was achieved by the heuristic model (99.45%) when using title text, which was followed by GB (98.67%). When using abstract and combined title and abstract texts, the BERT-based models showed similar performance to the conventional and CNN models.

**Table 4:** Summary of evaluation results for predicting non-RCTs with a heuristic model, three conventional machine learning models (LR, SVM, and GB), a neural networks model (CNN), and three BERT-based models (BERT, BioBERT, and SciBERT) for using (a) title, (b) abstract, and (c) combined title and abstract as input data. Bold indicates the best performance results among all the models. LR: Logistic Regression, SVM: Support Vector Machines, GB: Gradient Boosting, CNN: Convolutional Neural Networks.

**Method**	**Model**	**Accuracy (%)**	**Precision (%)**	**Recall (%)**	**F1 (%)**
Heuristic	Heuristic	87.97	87.28	**99.45**	92.97
Conventional Machine Learning	LR	91.54	92.48	97.34	94.85
	SVM	91.80	92.66	97.47	95.01
	GB	89.60	89.43	98.67	93.82
Neural Networks	CNN	92.01	93.12	97.19	95.11
BERT-based	BERT	92.05	93.96	96.25	95.09
	BioBERT	**92.96**	**94.86**	96.42	**95.63**
	SciBERT	92.87	94.73	96.45	95.58
(a) Title texts					
**Method**	**Model**	**Accuracy (%)**	**Precision (%)**	**Recall (%)**	**F1 (%)**
Heuristic	Heuristic	90.66	92.15	96.54	94.30
Conventional Machine Learning	LR	94.77	95.63	97.93	96.77
	SVM	95.12	95.96	98.02	96.98
	GB	94.98	95.71	**98.12**	96.90
Neural Networks	CNN	95.40	96.61	97.67	97.14
BERT-based	BERT	96.02	96.99	98.06	97.52
	BioBERT	96.23	**97.42**	97.88	97.65
	SciBERT	**96.28**	97.32	98.05	**97.68**
(b) Abstract texts					
**Method**	**Model**	**Accuracy (%)**	**Precision (%)**	**Recall (%)**	**F1 (%)**
Heuristic	Heuristic	91.78	93.49	96.44	94.94
Conventional Machine Learning	LR	94.93	95.71	98.06	96.87
	SVM	95.33	96.09	98.15	97.11
	GB	95.27	95.95	**98.22**	97.08
Neural Networks	CNN	95.29	95.98	**98.22**	97.09
BERT-based	BERT	96.27	97.30	98.05	97.68
	BioBERT	**96.37**	**97.55**	97.91	**97.73**
	SciBERT	96.36	97.42	98.05	**97.73**
(c) Combined (title + abstract) texts					

### 3.2 Sample size change

We ran the second experiment with abstract texts where sample size changed to see if there is much difference in model performance given sample data. A dataset of 100,000 instances randomly selected from the collection was used for this experiment. After splitting the dataset into training, validation, and testing subsets with the ratio of 8:1:1, abstract samples were selected from the training set from 1,000 to 10K with an increment of 1,000 (i.e., 1000, 2000, 3000, …, 9000) and then with an increment of 10k from 10K to 80K (i.e., 10K, 20K, 30K, …, 80K). To mitigate the variability of sampling, we repeated the random sampling for each sample size ten times and calculated the average scores of results. GB and SciBERT were selected as representative conventional and BERT models, respectively, for training and test because they showed high F1 scores when using abstract texts as input data. Only the size of training sample changed and all the other settings such as hyperparameters, optimization, etc. remained the same to see changes in model performance based on the data size change.

**Fig 1:**
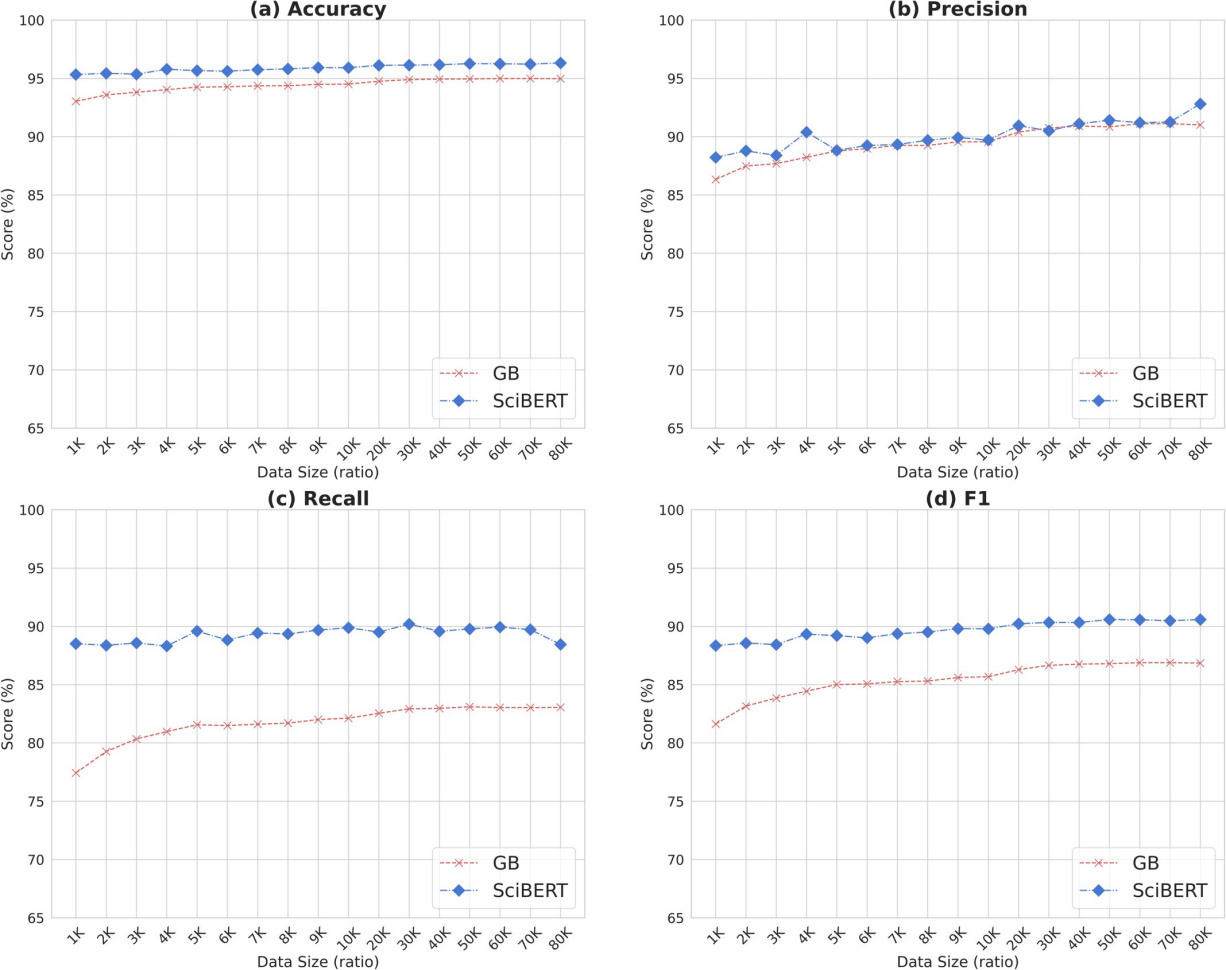
Distribution of average scores of four evaluation metrics for a conventional machine learning model (GB: Gradient Boosting) and BERT model (SciBERT) based on changes in data size.

Fig 1 shows the distribution of average scores of four evaluation measures–accuracy, precision, recall, and F1—for testing GB and SciBERT by sample size. The main finding is that the change in performance is more salient for GB than SciBERT. According to the figure, the GB model improved when provided with additional training data, but the rate of improvement decreased when the sample size reached 10,000 abstracts. For SciBERT, however, there is little change in the graph curve for any of the evaluation metrics, which indicates that sample size does not affect the performance of the BERT model and quite consistent levels of performance can be obtained with small samples.

It is worth noting that the BERT model showed much higher performance (88~90%) in terms of recall than the traditional machine learning model (77~83%) which was negatively impacted by small-sized samples. Even when the sample size increased, the gap in the performance of the two models did not decrease much. In summary, the BERT model maintained consistent result scores regardless of sample size for the evaluation metrics except precision, for which shallow fluctuation was shown with small portions of data. This demonstrates that the BERT-based models learn well based on small-sized training data, producing stable performance.

### 3.3 Balancing classes

Classes in our dataset are not balanced: the ratio of RCTs and non-RCTs is set to 1:4. To check whether any label imbalance affects performance differences between models, we balanced the class ratio to 1:1 and trained the two models (GB and SciBERT) based on the balanced data with different training data sizes. We re-used the subsets of training, validation, and test data from the Section 3.2 Sample size change for this experiment. To balance the label classes, we employed an under-sampling approach using the imbalanced-learn library (version 0.8.1) (https://imbalanced-learn.org/stable/), a python package used for resampling to handle imbalanced classes, to make the size of non-RCT labels equal to that of RCT labels. Next, training data was randomly sampled to produce different sizes of training data starting with a ratio of 0.1. The ratio changed by 0.05 and the two selected models were trained on each subset of balanced data. Note that only the size of training data changed and all the other settings in the models such as hyperparameters remained the same. To reduce the impact of sampling variability on measures, the per-ratio sampling was conducted ten times each and its performance scores were averaged.

**Fig 2:**
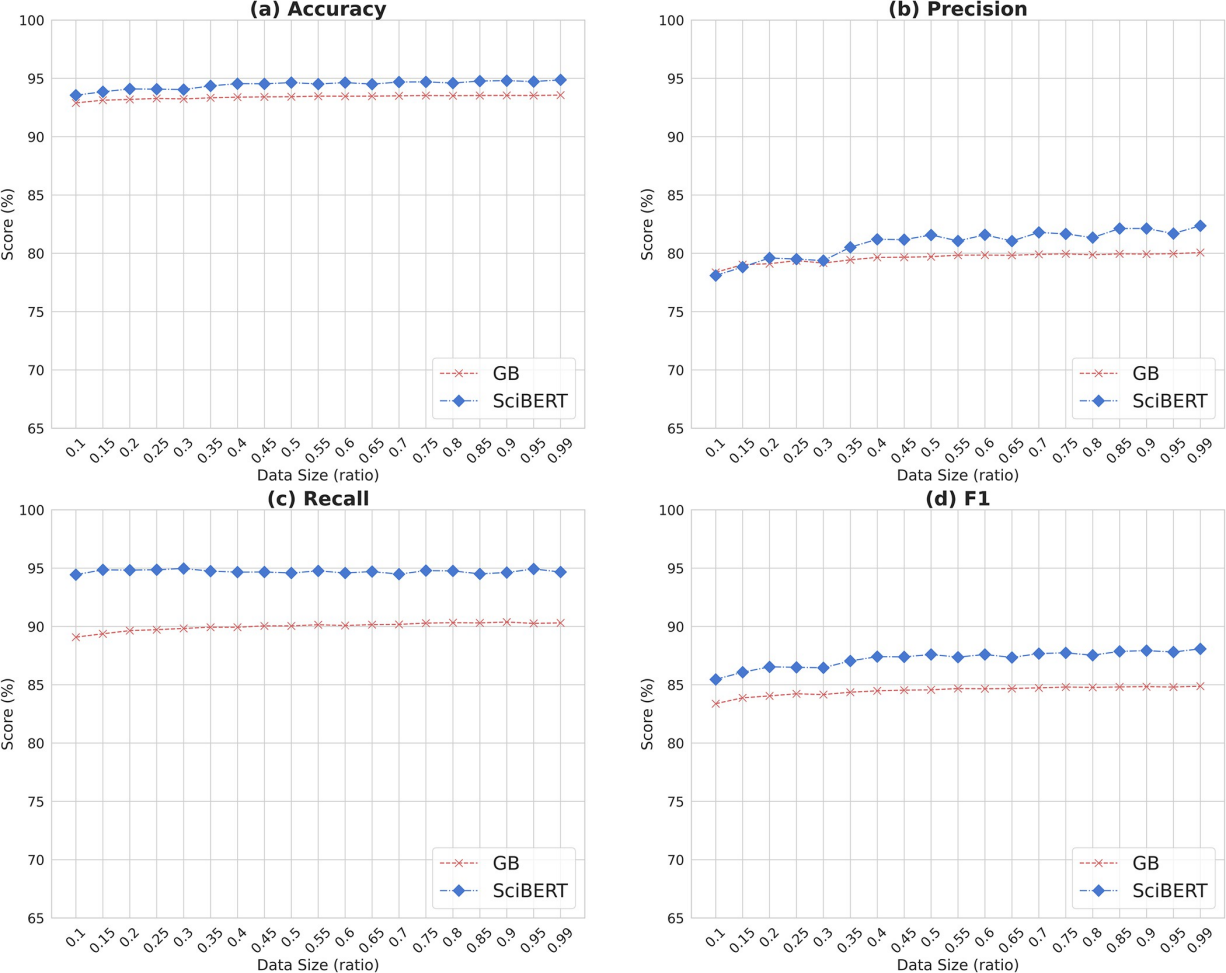
Distribution of average scores of four evaluation metrics for a conventional machine learning model (GB: Gradient Boosting) and a BERT-based model (SciBERT) based on training data size change after balancing class to 1:1.

Fig 2 reports the distribution of mean scores of four evaluation measures–accuracy, precision, recall, and F1—by sample size for a classical machine learning model (GB) and a BERT-based model (SciBERT) trained on the balanced data. Overall, SciBERT produced higher recall scores than GB consistently across a variety of balanced data sizes. As training data increased in size, precision by SciBERT also increased but recall scores were quite steady. Meanwhile, the GB model’s scores showed less variation but underperformed compared to SciBERT. Compared to the imbalance scenario above, recall scores by both GB and SciBERT were higher while precision scores were not. In addition, the performance gaps between the two tested models were narrower when data were balanced than when imbalanced.

## 4. Discussion

Conventional ML classifiers, CNN, and BERT-based models have been employed to classify MEDLINE data (title, abstract, combined title and abstract) for RCTs. In this work, we used three BERT-based models (BERT, BioBERT, and SciBERT) to achieve a maximum F1 score of 90.85% in predicting instances of RCT publication correctly. Our results show that the performance of BERT-based models is superior to those of traditional classifiers and the CNN model. The performance gains of BERT-based models were substantial in recall and F1. Such performance differences might be due to the fact that the BERT-based models used language models already pre-trained on large amounts of data. For example, the BERT version used for our study was trained on the corpus that includes more than 800M words. The language models trained on the massive text data were transferred to our study’s modeling to account for a word’s context in the text data we used. While previous methods of word-embedding would return the same vector for a word no matter how it is used, the BERT-based models return different vectors for the same word depending on the words surrounding it. This consideration of word context seems to enable the BERT-based classification models to find many true cases of RCT texts (which increased recall) that were missed by the conventional ML algorithms and CNN.

In addition to their superior performance, BERT-based models provided procedural benefits in the implementation of text classification tasks. In other words, there was no need for any feature extraction and selection before feeding into a classifier. This simplified the pre-processing steps used in traditional machine learning approaches. It is also worth noting that the time required to run SVM was excessive although it showed the best performance among the conventional machine learning models for all three types of text. For example, it took over 85 hours for training and testing SVM with abstract text only. On the contrary, it took only 15 hours to run the SciBERT model on the same data, which not only surpassed the results of SVM but also produced the best results among all six models in terms of F1 score. The total time to train and test all three BERT-based models with the three types of text was about 102 hours. This implies that using a BERT-based model not only gives better results, but also saves time and computing costs when analyzing big data on cloud computing.

## 5. Conclusion

Randomized controlled trials (RCTs) play a major role in producing research and informing practices by providing an approach to examine the effect of interventions on specific outcomes such as death or the recurrence of disease. The demand for highly accurate retrieval of scientific articles has grown and correctly identifying all the published RCTs in a given domain is not a simple task. This paper presented Bat4RCT, a combination of dataset and strong baseline method for RCT classification tasks applying the use of BERT-based models which transfers language models trained on massive text data. This study is the first to introduce the approach of using pre-trained language modeling techniques in classifying RCT texts. Our motivation came from the various reports that BERT-based models have outperformed conventional machine learning algorithms and neural network models in text classification. Three BERT-based models (BERT, BioBERT, and SciBERT) were implemented and the results were compared to the ones obtained from a heuristic approach, three classical machine learning classifiers, and CNN. All the models trained and tested were integrated into our proposed system, Bat4RCT.

Our main finding is that the BERT-based models showed much higher recall scores for any type of texts as input data compared to the heuristic and other machine learning models used in previous studies. The best performance was achieved by the BioBERT model when trained on both title and abstract texts, with the F1 score of 90.85%. SciBERT showed a slightly higher F1 score than BioBERT when using abstracts only. However, BioBERT was the winner of all types of texts when considering the recall score. One practical implementation scenario of our method would be to fine-tune our models to produce classification results with probability scores and pass cases with scores below a certain threshold to human annotators to classify the texts describing RCTs accurately. Bat4RCT, which includes shared code and a dataset, can be reused for validation of our results and serve as a strong baseline to improve existing RCT classification techniques and develop novel methods in future research.

A major limitation of our study is that the size of the used dataset is limited although it is comparable to the prior study using the largest training and test data [2]. Further improvement can be explored by using larger input datasets than used in this work. In future work, we plan to expand our study to include a larger size of data and to leverage automated methods for detecting RCTs in the biomedical literature. A second limitation of our study is that we only examined three pre-trained models and did not utilize other deep learning techniques such as Recurrent Neural Networks (RNN). Our work is expected to motivate and guide future studies that use deep learning approaches in the field of RCT classification in biomedical research.
